# Editorial: Smart Tools for Caring: Nanotechnology Meets Medical Challenges

**DOI:** 10.3389/fbioe.2019.00011

**Published:** 2019-01-30

**Authors:** Giada G. Genchi, Gianni Ciofani

**Affiliations:** ^1^Smart Bio-Interfaces, Istituto Italiano di Tecnologia, Pontedera, Italy; ^2^Department of Mechanical and Aerospace Engineering, Politecnico di Torino, Turin, Italy

**Keywords:** smart materials, remote stimulation, drug delivery, tissue engineering, clinical translation

This Research Topic of Frontiers in Bioengineering and Biotechnology-Nanotechnology stems from the international workshop “Smart tools for caring: Nanotechnology meets medical challenges” held in Pontedera (Italy) on March 2, 2018 with the goal of promoting a multidisciplinary discussion on the most innovative techniques and materials with strong therapeutic potential in human healthcare. The issue presents 14 selected, peer-reviewed contributions (1 Brief Research Report, 7 Mini-Reviews, 1 Opinion, 3 Original Researches, 3 Reviews) from different research fields, ranging from materials science, to biotechnology, physics, and engineering.

In agreement with the scopes of the workshop, this issue reviews general topics of crucial importance in the design of materials for interaction with biological systems, most notably mechanical properties, surface features (including roughness, porosity, and patterning), and their spatiotemporal variation (Cimmino et al.; Palamà et al.). The synthesis and fabrication of innovative materials are presented with focus on the modulation of cell responses in events like cell adhesion, proliferation, and differentiation (Bandiera et al.; Denchai et al.; Şen et al.; Suarato et al.). In this concern, instrumental techniques for characterization of bio-/non bio-interactions (Arai and Ferdinandus; Fujita et al.; Tonda-Turo et al.), light-based methods for remote stimulation of materials and their anatomical targets (Bossio et al.; Polino et al.), and complex approaches for diagnosis and treatment of pathological conditions through genetic manipulations of somatic cells, nanomaterials, and robotic devices (Cervadoro et al.; Robinson et al.; Soto and Chrostowski) are presented.

More in detail, the importance of material properties like stiffness is discussed with relevance to the fabrication of three-dimensional constructs of glioblastoma multiforme, with the final goal of elaborating reliable therapies against this aggressive tumor of the central nervous system (Palamà et al.). Along with stiffness, other biochemical and biophysical signals like ligand exposure and topography of the materials are thoroughly reviewed, and the significance of their time- and space-variant patterns in response to external stimulations (light, temperature, electricity, or even enzymes and cells) is discussed in relation to the elaboration of specific protocols for exposure of cells to dynamic bioinstructive signals (Cimmino et al.).

The importance of the use of polypeptides (for instance collagen, fibroin, and keratin), polysaccharides (like hyaluronic acid, chitosan, and alginate) or even their mixtures of natural origin (such as in mycelia with controlled composition based on the fungal feeding substrate) is stressed as an environmental sustainable strategy for the preparation of biodegradable materials, especially in the context of wound healing (Suarato et al.).

In this Research Topic, the fabrication of novel materials with controlled architecture is addressed with electrospinning and wet chemistry techniques. Corroborated by computational models that predict and correlate surface properties with biological responses, electrospinning is reviewed as an effective technique for micro- and nanopatterning of materials to impart biologically relevant cues (under the form of fiber orientation, roughness, and density) to tune cell adhesion, proliferation, and differentiation (Denchai et al.). Electrospinning is also used as a technique for the fabrication of hybrid scaffolds with thermoresponsive hydrogels (based on recombinant elastin-like polypeptides) in view of drug loading and release in elastolytic conditions, such as those associated to wound healing (Bandiera et al.). One step synthesis of hexagonal boron nitride nanomaterials (hBNs) from different boron sources and in the absence of catalysts is also described for possible application in cancer treatment. In particular, evidences on hBN stability and degradation under both oxidative and hydrolytic conditions are reported, supporting the use of colemanite as a boron source (Şen et al.).

Microscopy techniques for investigating biological events with intracellular compartment resolution are discussed: basic indications of the choice and application of fluorescent probes are presented with relevance to photothermal and photodynamic therapeutic approaches (Arai and Ferdinandus). Moreover, methods based on probes (genetically encoded fluorophores, chemical fluorescent molecules, and other nanomaterials) are reviewed along with purely optical, probe-less methods (Fujita et al.). Among the techniques for characterization of bio-/non bio-interactions, quartz crystal microbalance is described as a tool for dynamic investigation of chemical, mechanical, and electrical properties of surfaces interacting with cells and of biological response to biomedical devices (Tonda-Turo et al.).

The activation of smart materials on their target sites by non-invasive approaches is here discussed with a focus on phototransduction methods based on photovoltaic devices or photocatalytic nanoparticles. Photovoltaic devices are presented for stimulation of electrogenic cells in the retina and for promotion of non-electrogenic cell proliferation, such as during angiogenesis and wound healing (Polino et al.). Photocatalytic nanoparticles based on poly(3-hexylthiophene) with semiconductive properties are exploited for modulation of calcium influxes in HEK-293 cells through the generation of reactive oxygen species under visible light irradiation, denoting potential for cell behavior tuning with high spatio-temporal resolution (Bossio et al.).

This Research Topic also includes a concise review of innovative materials with theranostic potentiality for modulation of cell response in terms of inflammation control, immune system targeting, and treatment of atherosclerosis (Cervadoro et al.). An innovative approach for genetic reprogramming of somatic cells (astrocytes) into neurons with a non-viral method (based on a fusion protein containing a transcription factor and a protein transduction domain, in association to SMAD, Notch, and histone deacetylase inhibitors) is presented for possible treatment of central nervous system injuries (Robinson et al.). The need of considering emerging clinical translation and potential commercial use of micro- and nanorobotic devices is also thoroughly reviewed: even in this case, the discussion stresses on fine spatiotemporal control of activities such as delivery of drugs, imaging agents or cells, and biopsy collection. The discussion is completed by considerations on experimental methodology reproducibility and standardization requisites, on intellectual property protection, on device fabrication and on the implementation of innovative devices in current therapeutic protocols for the elaboration of concrete translational medicine approaches (Soto and Chrostowski).

To conclude, we hope this Research Topic will represent a useful reference of easy consultation for those readers interested to gather concise information on the state of the art, as well as to understand the necessary directions of multidisciplinary research on innovative materials and related topics of high relevance to human healthcare for the immediate future.

## Author Contributions

All authors listed have made a substantial, direct and intellectual contribution to the work, and approved it for publication.

### Conflict of Interest Statement

The authors declare that the research was conducted in the absence of any commercial or financial relationships that could be construed as a potential conflict of interest.

